# Gold, Gold-Beating, Compounds and Alloys

**Published:** 1853-01

**Authors:** R. N. Wright


### ARTICLE VIII.

### *Gold, Gold-beating, Compounds and Alloys.* By R. N. Wright, M. D.

Gold is one of the most ancient metals of which we have any authentic record, and one of the few metals almost univerally found native. There is scarcely a region of the globe, in which it does not occur to some extent, greater or less, according to circumstances. The largest amount obtained, until within a few years, was from the neighborhood of the Ural mountains, in the Russian possessions, and in certain sections of the United States and South America. More recently, however, immense supplies have been obtained both from California and Australia. It is the metal known to the ancients as "sol," (the sun of the metals,) and the only metal possessing the bright yellow colors.

Gold, as found, whether mixed with other substances or comparatively pure, exhibits very different shades of color, varying from a deep orange to a light lemon yellow, and though it is commonly found in primary formations, yet, occurs in considerable quantities mixed with the alluvial soil, deposited in the beds and on the shores of mountain streams.

Various methods have, from time to time, been devised for freeing gold from impurities, (whether earthy or metallic,) with which it is found in combination. A method most commonly practiced at one time was amalgamation, a process liable to many objections. Another process consists in separating it from copper or silver, by means of nitric acid.

The process for the purification of gold proposed by Graham, page 464, is as follows: "The alloy containing the gold, is dissolved in a mixture of two measures of hydrochloric and one of nitric acid, a mixture which, from its application to dissolve gold, has been termed 'aqua regia.' The solution of gold is evaporated by a water bath until acid vapors cease to be exhaled, it is then dissolved in water and hydrochloric acid is added to it. On adding protosulphate of iron to this solution, the gold is wholly precipitated, as a brown or brownish yellow powder, quite destitute of metallic lustre, which, however, appears when the powder is rubbed. The protosulphate of iron is, at the same time, converted into persulphate and perchloride."

Blande's method, as described page 972, is as follows: "Dissolve one part by weight of standard gold, in three of nitrohydrochloric acid, (composed of one part by weight of nitric and two of hydrochloric acid,) evaporating the solution to dryness, (by a gentle heat towards the end of the process,) redissolving the dry mass in distilled water, filtering and adding to it a solution of protosulphate of iron. A black powder falls, which after having been washed with dilute hydrochloric acid and distilled water, affords, on fusion with a little borax, or other suitable flux, a button of pure gold." Either of the above processes will be found on careful repetition to answer well.

Gold is malleable and ductile to a remarkable extent, and is thus made one of the most useful metals in the arts for covering surfaces, with a material which will not tarnish on exposure to the air; it is also now very extensively used in the various operations of dentistry, (a description of which would be foreign to the purpose of this article.) Gold may be hammered by a process termed "gold-beating," into leaves, not exceeding the 280,000th of an inch in thickness, in which condition, they are diaphanous, admitting to the eye when held before it, a greenish light; and one grain may be extended into a wire five hundred feet in length.

We proceed to give below, (for the benefit of such as may be interested,) an account of the procees of "gold-beating," copied from "Tomlinson's Cyclopœdia of the Useful Arts," page 793.

"The gold employed by the 'gold-beater' should be pure; but various colors are obtained by alloys with silver or with copper, in different proportions. The pure gold, or the alloy, is prepared for the gold-beater by melting in a crucible, and casting into flat oblong ingots, each about $\frac{3}{4}$ of an inch wide, and weighing two ounces, each ingot is then formed into a riband by passing it between two rollers of polished steel, and this laminating process is continued until the ingot is spread out to a surface of 960 square inches of the thickness of rather more $\frac{1}{800}$ of an inch. The riband of gold is annealed or softened in the fire, and cut up into pieces of the size of a square inch, and 150 of these are placed by means of wooden pliers, between an equal number of leaves of vellum, each square of gold occupying the centre of each leaf of vellum. A parchment case, open at both ends, is drawn over this tool, or kutch, as the packet of vellum leaves is called, and this is enclosed in a second similar case, so as to cover the edges left exposed by the first case. This packet is then beaten with a sixteen pound hammer upon a smooth block of marble, strongly supported from below, and surrounded on three sides by a raised ledge of oak; the front edge is open, and has a kind of leather apron attached to it for catching fragments of gold that may escape in the subsequent operations. The elasticity of the packet causes the hammer to rebound and thus lightens the labor of the operator, and enables him to apply his blows with regular effect; every now and then, in the interval between two blows, he turns the packet over to distribute the force equally, and he occasionally bends the packet to and fro to overcome any slight adhesion between the gold and the vellum, and at intervals he opens the packet to see that the work is satisfactory, and also to re-arrange the relative positions of the squares of gold by placing those near the surface in the centre, and placing these near the surface. The beating is continued until the one inch squares are spread out into four inch squares. The packet is then opened, and each piece of gold is taken out, placed on a cushion, and cut into four pieces with a knife. This increases the 150 pieces to 600, and these are put between the leaves of another tool, called a shoder, made of gold-beater's skin. The packet is enclosed in parchment, and beaten with twelve-pound hammers as before. The squares of gold are again spread out to nearly the area of the gold-beater's skin. The packet is again opened, and the leaves of gold are again cut into fours, and each quarter is placed between two leaves of membrane as before. The gold is in this case divided by means of the smooth edge of a strip of cane, since it has a tendency to adhere to a steel knife.

"The squares of gold, now increased to 2,400, are separated into three parcels of 800 each; the squares of each parcel are again separated by gold-beater's skin confined in the parchment cases, and beaten as before. These squares of gold-leaf expand for the third time nearly to the size of the leaves of membranes, and have at length attained the required degree of tenuity. The process of attenuation can be carried beyond this, but the gold is apt to tear, and the process requires great extra care. The three beatings and two quarterings, expand the gold to an area about 190 times greater than it had in the form of a riband, and 100 square feet of it weigh only an ounce. After the last beating, the thin leaves are taken up one at a time by means of a pair of long pincers, made of wood, and being placed on a cushion, are blown out flat by the mouth, an operation requiring considerable skill. Broken or injured leaves are rejected, but those which are perfect have the ragged edges cut off, which reduces their dimensions to about 3¼ inches square. Twenty-five of these leaves are placed between the folds of a paper book, the surfaces of which have been rubbed with red chalk, to prevent the gold from adhering, and in this form gold leaf is sold."

It was at one time supposed, that gold did not unite under any circumstances with oxygen; the certain existence of two oxyds can now be demonstrated, and some chemists believe in the existence of a third or intermediate condition of oxydation.

The protoxyd of gold consists of gold 1 atom, oxygen 1 atom, and is thus represented, (au + o.) It can most readily be prepared, by adding to the protochloride of gold a cold solution of potash. After obtaining the result, it is best to wash it with water, at a temperature not exceeding 100°; the reason of which is, that by carrying the heat much beyond this we may reduce some portion of the oxyd into metallic gold and peroxyd. There is also another precaution necessary, excess of the alkali will sometimes dissolve it and hence it must be used sparingly and carefully. When obtained by the above process, we have a product of an olive color.

The peroxyd of gold may be obtained by a number of processes; we will, however, mention but two, viz. those of Turner (proposed on the authority of Dr. Wagner, of Perth, in Hungary,) and that of Pelletier.

The former proposes to "dissolve 1 part of gold in the usual way, renders it neutral by evaporation, and redissolve in 12 parts of water; to the solution, add 1 part of carbonate of potash, dissolved in twice its weight of water, digest at about 170°; carbonic acid gradually escapes and the hydrated peroxyd of a brownish red color subsides, after having been well washed, it is dissolved in colorless nitric acid of the specific gravity 1-4 and the solution decomposed by admixture with water; the hydrated peroxyd is thus obtained quite pure, and is rendered auhydrous by exposure to a temperature of 212°. In this auhydrous state, it is nearly black, insoluble in water, and decomposed either by exposure to solar light or heat. It is soluble in nitrohydrochloric acid, forming the common solution of gold, and it dissolves in sulphuric and in nitric acids, but the affinity is here so weak that the solutions are decomposed by the action of water and yield no saline compound when evaporated with the utmost caution.

These properties led Pelletier to examine the action of alkalies upon this oxyd, and he found that digested in a solution of caustic potash, it was dissolved; it also combines with baryta, and in these cases, apparently, plays the part of a weak acid. Boiled with chloride of potassium or sodium, a yellow solution results, which is alkaline and contains chloride of gold and aurate of soda or potassa—This oxyd consists of*

| | |
|---|---|
| Gold, . . . . . | 1 |
| Oxygen . . . . . | 3 |
| | — |
| Peroxyd of gold, . . | 1—(au + O3.) |

In describing the oxyds of gold, we have reserved the deutoxyd, (already alluded to,) until now, because, so much doubt exists as to the reality of such a substance.

*** The above is from Brandi, page 973.**

The result produced by the passage of a powerful electrical current through gold well beaten, is a purple substance, which has been called an oxyd of gold, and supposed to be intermediate between the protoxyd and peroxyd. It is, however, no doubt, simply gold in a state of fine comminution, for, we cannot at present say, we have any tests which determine its absolute existence as an oxyd of gold.

**(To be continued.)**

